# Radiation therapy in mycosis fungoid patient

**DOI:** 10.11604/pamj.2019.33.227.17701

**Published:** 2019-04-17

**Authors:** Madiha Chellakhi, Ilham Khalfaoui, Nadia Benchakroun, Zineb Bouchbika, Hassan Jouhadi, Nezha Tawfiq, Souha Sahraoui, Abdellatif Benider, Nabila Chellakhi, Asmaa Quessar

**Affiliations:** 1 Centre Mohammed VI de Traitement du Cancer, CHU Ibn Rochd, Casablanca, Maroc; 2 Service d’Hématologie et d’Oncologie Pédiatrique Hopital 20 Aout 1953, CHU Ibn Rochd, Casablanca, Maroc

**Keywords:** Mycosis fungoid, radiotherapy, skin, T-cell lymphoma

## Abstract

Mycosis fungoid (MF) is a non-Hodgkin's T-cell lymphoma determined by primary cutaneous involvement. It is a slow-progressing chronic indolent disease characterized by atypical T-cells with a cerebral nucleus. Management of this disease depends on the stage and is based essentially on the systemic treatment. Radiotherapy intervenes in case of localized or extended tumor, indeed, the radiosensibility of this tumor, like any other hematological affection, makes it possible to obtain a high rate of response. Clinical case: we report the observation of a 46-year-old patient followed since 2012 for mycosis fungoid revealed by a papullo-squamous lesion located at the level of the right lumbar fossa. The diagnosis was confirmed by cutaneous biopsy, showing the presence of T lymphocytes expressing CD2, CD3, CD4, CCR4, CD45RO markers. Initial assessment included a thoraco-abdominal pelvic CT, which was normal, an accelerated sedimentation rate at the 1^st^ hour, a high C reactive protein (CRP), the electrolytic, renal, hepatic status and the hemogram were normal. Patient received 6 courses of chemotherapy according to the COPP protocol with a decrease in the size of the lesion estimated at 40%. A norm fractionated radiation therapy was delivered at the dose of 36Gy. The evolution was marked by a complete remission, maintained after 6 months of the treatment. Mycosis fungoid is a rare disease, whose management must be discussed in a multidisciplinary team. Radiotherapy remains an interesting option for all stages, but has to be validated in largest studies.

## Introduction

Fungoid mycosis is a malignant T-cell proliferation that invades the skin. It is known by its radio-sensitivity. However, some patients are initially treated with chemotherapy and the use of radiation is only palliative in case of symptomatic lesions or increasing volume [1]. Radiation therapy is a very important therapeutic option for both localized and extensive lesions. The rarity of this disease did not allow the realization of randomized studies evaluating the indications of the treatment by radiotherapy. This case of mycosis fungoid that we report is illustrated by a review of the literature and will highlight the place of radiotherapy in the treatment of this hematological disease.

## Patient and observation

**Figure 1:**
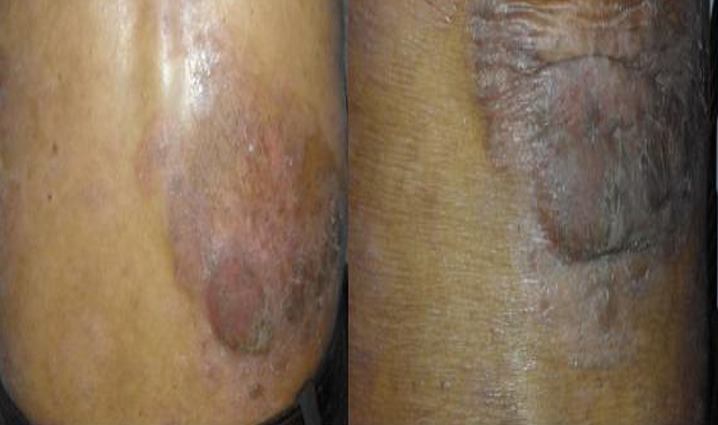
Squamous papular lesion of mycosis fungoides in the lumbar region refractory to conventional chemotherapy

**Figure 2:**
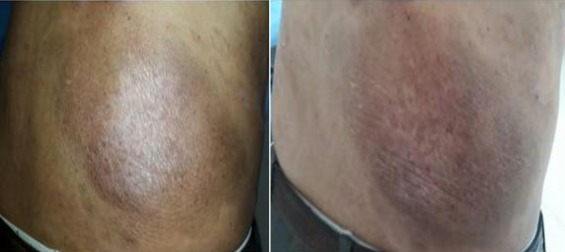
The right lumbar lesion of mycosis fungoides after external beam radiation therapy 18x2Gy, response maintained after 6 months

We report the case of a 46-year-old patient with no specific history followed since 2012 for mycosis fungoid revealed by an erythematous and prurigino-papulo-squamous elementary lesion located in the right lumbar fossa (Figure 1) which was progressively increasing in size. Symptomatic treatments have, in vain, been administered such as emollients, keratolytics, systemic and topical corticosteroids. Cutaneous biopsy showed the presence of atypical T lymphocytes expressing the markers CD2, CD3, CD4, CCR4, CD45RO as well as the cytotoxic markers CD30 and TIA-1. The initial assessment included a thoracoabdominal pelvic CT scan, showing no visceral or lymph node involvement, an accelerated sedimentation rate at the 1^st^ hour, a high C reactive protein (CRP), the hemogram, renal and hepatic functions were also performed and were normal. After failure of previous therapy with PUVA-therapy and interferon-alpha, chemotherapy was introduced, of which the patient received 6 curses according to the COPP protocol with a decrease in the lesion's dimension estimated at 40%. Electron-beam radiotherapy at a dose of 36Gy in normo-fractionated (18x2Gy) was delivered, noting a clear regression of the extent and size of the lesion as early as the first week. The evolution was marked by a complete remission, maintained after 6 months of the end of radiotherapy (Figure 2).

## Discussion

Mycosis fungoide (MF) is a non-Hodgkin's T-cell lymphoma determined by a primary cutaneous involvement. It is a slowly progressing chronic indolent disease, characterized by the development of large lesions, or tumors on the skin due to the proliferation of atypical T cells [2]. The diagnosis of MF is often difficult, mainly because of the atypical clinical presentation at an early stage. Indeed, as the case for this patient, lesions can simulate psoriasis, atopic dermatitis or chronic eczema. The diagnostic procedure begins with the determination of history of the disease, the description of the dermatological lesions and in the next step, the skin sample for histological study. Malignant T cells with T-Helper memory are characterized by the following phenotype: CD2 +, CD3 +, CD4 +, CD5 +, CCR4 +, CD45RO +, rarely CD8 +, CD7- and CD26. T cells also express lymphocytic cutaneous antigen, TH2 cytokines and advanced cytotoxic markers; CD30 and TIA-1 [3]. MF is classified into 4 clinical stages according to the TNMB classification (tumor-node-metastasis-blood) that takes into account the extent of cutaneous involvement based on the body surface, the presence of nodal or visceral disease and the presence of sezary cells at peripheral blood level [4, 5]. MF is generally considered to be an incurable disease, but it should be noted that most patients have indolent forms classified stage IA or IB in 65 to 85% and are, therefore, long-term survivors [6, 7]. Management depends on the stage and is based on local, systemic treatments and radiotherapy. Topical skin treatments mainly include systemic or topical corticosteroids, such as betamethasone dipropionate 0.05%, they allow a complete and partial response rate of 60-65% and 30% in case of T1 and 25 and 57% in the T2 stage, respectively [8]. Generalized skin treatment is mainly represented by phototherapy or PUVA therapy (Psoralen plus ultraviolet A light therapy) which is indicated in patients with more extensive lesions [9].

Systemic treatments include: interferon α, systemic retinoids and histone deacetylase inhibitors (HDAC). These treatments are preferred in non-responders to local therapies, compared to standard chemotherapy and achieve an objective response rate of up to 90% [10, 11]. Conventional chemotherapy can be used, from the outset, in case of locally advanced disease or second line in early stages refractory to local therapies and systemic biological treatments, such was the therapeutic behavior in the case reported. Cytotoxic drugs may be used alone or in combination, including methotrexate, chlorambucil, vincristine, doxorubicin, cyclophosphamide, etoposide and gemcitabine and nucleoside analogues. The different combinations are associated with a high level of objective response to mono-chemotherapy [12, 13]. The radiotherapy intervenes in case of localized tumor or extended, indeed, the radio-sensitivity of this tumor, like any other hematological affection, makes it possible to obtain a high rate of response. According to NCCN the effective dose varies between 12 and 36Gy [14]. Radiotherapy can be delivered on lesions of variable extent with curative or palliative aims. In case of localized disease or single lesion, a 100% complete response rate at 2 months has been reported, with an average response time of more than 40 months [15]. Low-dose palliative electron-therapy (4Gy in 2 fractions) can be delivered in cases of refractory generalized disease on symptomatic lesions. The complete response rate can reach 95% in the case of small tumors (3cm) with possibility of reirradiation or irradiation of newly emerging lesions [16, 17]. Whole body electron irradiation at a dose of 30Gy, 2Gy/J, 4d/week can be proposed in any stage with a high rate and duration of response [18]. A retrospective study of 141 patients treated with Whole body irradiation with electrons, complete remission was obtained in 87.5% of T1-stage tumors and in 84.8% of T2-stage tumors. The disease-free survival and 5-year overall survival rates were 50% and 65%, respectively [19]. MF is an indolent disease whose main prognostic factors are age at diagnosis, extent and type of skin lesion, stage, extra-cutaneous extension and the presence of atypical cells in peripheral blood [20].

## Conclusion

The treatment of mycosis fungoides is a challenge. Indeed, this disease must be managed by a multidisciplinary team. Radiation therapy remains an important therapeutic option that can intervene in all stages of the disease however, this option requires validation by more large studies.

## Competing interests

The authors declare no competing interests.
